# The Link between Stroke Risk and Orodental Status—A Comprehensive Review

**DOI:** 10.3390/jcm11195854

**Published:** 2022-10-02

**Authors:** Shahriar Shahi, Mehdi Farhoudi, Solmaz Maleki Dizaj, Simin Sharifi, Saeed Sadigh-Eteghad, Khang Wen Goh, Long Chiau Ming, Jagjit Singh Dhaliwal, Sara Salatin

**Affiliations:** 1 Dental and Periodontal Research Center, Tabriz University of Medical Sciences, Tabriz 5166/15731, Iran; 2 Neurosciences Research Center (NSRC), Tabriz University of Medical Sciences, Tabriz 5166/15731, Iran; 3 Faculty of Data Science and Information Technology, INTI International University, Nilai 71800, Malaysia; 4 PAP Rashidah Sa’adatul Bolkiah Institute of Health Sciences, Universiti Brunei Darussalam, Gadong BE1410, Brunei

**Keywords:** stroke, brain, periodontitis, gingivitis, infection, oral and dental health

## Abstract

One of the primary causes of disability and mortality in the adult population worldwide is stroke. A person’s general health is significantly impacted by their oral and dental health. People who have poor oral health are more susceptible to conditions such as stroke. Stroke risk has long been linked to oral and dental conditions. The risk of stroke and its cost impact on the healthcare systems appear to be significantly reduced as a result of the decline in the incidence and prevalence of oral and dental illnesses. Hypothetically, better management of oral hygiene and dental health lead to reduced stroke risk. To the authors’ best knowledge, for the first time, the potential link between dental health and stroke were cross-examined. The most typical stroke symptoms, oral and dental illnesses linked to stroke, and the role of oral healthcare professionals in stroke prevention are revealed. The potential mediating processes and subsequent long-term cognitive and functional neurological outcomes are based on the available literature. It must be noted that periodontal diseases and tooth loss are two common oral health measures. Lack of knowledge on the effects of poor oral health on systemic health together with limited access to primary medical or dental care are considered to be partially responsible for the elevated risk of stroke. Concrete evidence confirming the associations between oral inflammatory conditions and stroke in large cohort prospective studies, stratifying association between oral disease severity and stroke risk and disease effects on stroke survival will be desirable. In terms of clinical pathology, a predictive model of stroke as a function of oral health status, and biomarkers of systemic inflammation could be useful for both cardiologists and dentists.

## 1. Introduction

The burden of stroke-related disability and death has been rising globally to this point [1,2]. However, there are currently no viable treatments for stroke, in part because it is difficult to deliver medications to brain regions that are affected. The risk factors for stroke must therefore be addressed. Studies that looked at a wide variety of characteristics, including health condition, genetic markers, and lifestyle, have verified a number of risk factors for stroke. Numerous epidemiological studies [3,4] have examined the relationship between poor dental health and an increased risk of stroke as one of the lifestyle-related variables. A mouth that has clean teeth, healthy gums, strong teeth, and a clean tongue demonstrates general good health and hygiene.

The major dangers to oral health are infectious illnesses and oral cancers [5]. Dental infections brought on by poor oral hygiene are among the most common chronic bacterial illnesses, frequently affecting those who avoid or put off going to the dentist. Gingivitis, periodontitis, and dental caries are among the most prevalent chronic orodental diseases in humans [6,7]. Dental infections can spread to nearby bone and soft tissues after starting inside a tooth or one of its supporting components. Lack of proper brushing and other oral hygiene practices promotes bacterial accumulation and plaque buildup on the teeth and gum line, which can cause significant inflammatory changes in the periodontal tissues. Therefore, maintaining good oral health is essential to avoiding plaque buildup that could result in gum disease or tooth decay.

**Figure 1:**
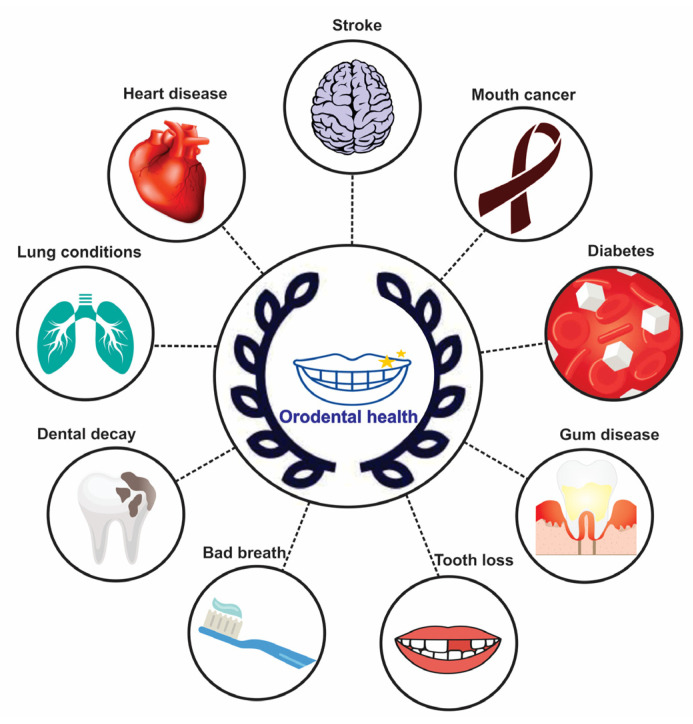
Link between oral health and general health.

Bacterial infections can cause serious inflammatory reactions and spread to other mouth regions if not treated right at once. Other organs and tissues may experience significant alterations as a result of oral inflammation, which is not just limited to the oral cavity. It has been observed that hypertension [8], heart disease [9], diabetes [10], pulmonary lung disease [11] and periodontal disease are all related to poor dental hygiene (Figure 1).

The significance of dental health in stroke has also been demonstrated by observational research. According to research, participants with periodontitis have a larger correlation with stroke than people who have coronary artery disease [12]. It has been demonstrated that several autoimmune diseases such as Lupus Erythematosus [13], psoriasis [14], and Sjogren’s Syndrome [15], predispose the patients to periodontal diseases. Several studies exhibit that poor oral hygiene has been associated with predisposition to metabolic disorders, which in turn can pose a risk of stroke [16,17]. The prevalence of oral disorders and the risk of stroke continue to be major global issues despite significant gains in the oral health of populations in many nations [18]. In this review, the potential link between dental health and stroke is examined. The most typical stroke symptoms, oral and dental illnesses linked to stroke, and the role of oral healthcare professionals in stroke prevention are all covered in the sections that follow.

## 2. Stroke

**Figure 2:**
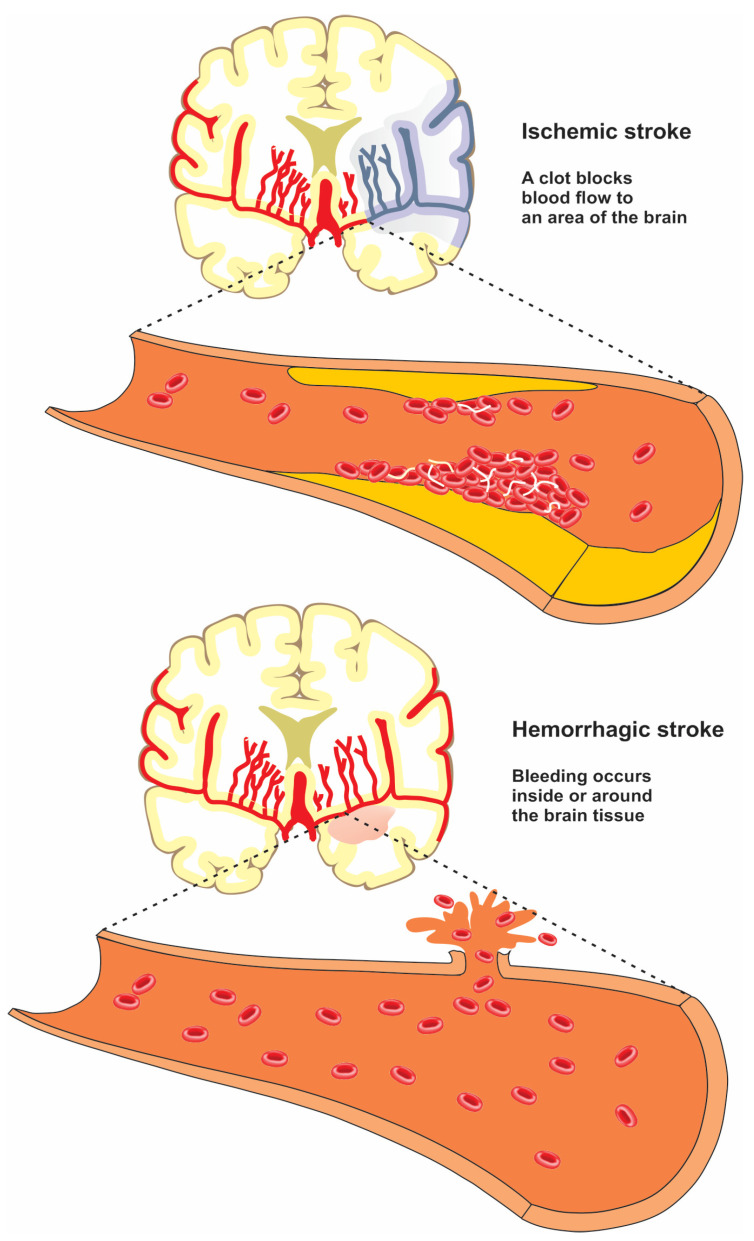
Difference between ischemic and hemorrhagic stroke.

Stroke is an acute condition characterized by the sudden loss of blood supply to a region of the brain, causing injury to the cells and tissues [19,20,21,22]. Stroke is typically divided into ischemic stroke and hemorrhagic stroke (Figure 2).

Ischemic stroke, also known as cerebral ischemia, occurs when there is a blockage in the blood supply to a part of the brain. Hemorrhagic stroke occurs when a blood vessel supplying the brain ruptures and bleeds [23,24]. Neurons begin to die within minutes of the onset of stroke because their oxygen supply is cut off. The brain damage caused by a stroke can impair a person’s fundamental bodily functions such as movement, memory, and speech [25,26].

Stroke is a multi-factorial disease caused by a mix of hereditary and environmental factors. This necessitates addressing and investigating the stroke risk factors [27]. Gender, age, diet, education, exercise, smoking, alcohol consumption, stress, diabetes, hypertension, cardiovascular disease, etc., are currently some of the most prevalent risk factors [28,29]. In addition to these traditional risk factors, epidemiological studies support the concept of a possible relationship between oral hygiene and stroke [30]. Recent results indicated that chronic exposure to common infections contribute to the risk of stroke, and that acute infections have been identified as potential stroke triggers [31]. Inflammation, an element of the body’s defense against surgery or injury, is significant in cardiovascular diseases such as heart attack or ischemic stroke [32]. Inflammation can be caused by several factors, including bacterial infection or surgery. In fact, poor oral hygiene can trigger a systemic inflammatory response from the invasion of oral bacteria and inflammatory mediators, and this systemic inflammation reaction can increase the risk of stroke [33].

Oral hygiene indicators can elicit the incidence of stroke in various ways and to varying degrees depending on the stroke subtype [34]. According to recent studies, ischemic stroke is more consistent with an infection theory, whereas hemorrhagic stroke is not often associated with dental or other infections and is significantly connected with hypertension [35,36].

Imaging investigations and physical examinations are frequently used to diagnose strokes. Studies on the prevention of stroke have begun to consider biomarkers, and several biomarkers have been identified [37]. Among them, it is commonly accepted that inflammatory biomarkers are associated with a higher risk of stroke etiology and outcome [38]. In the post-stroke brain, damage signals and danger-associated molecular patterns are released by dying neuronal cells. Inflammatory cytokines are then released by activated microglia and peripheral leucocytes, which enhance the inflammatory response. Studies have reported that elevated inflammatory markers are associated with the risk of stroke recurrence and less favorable outcomes [39,40]. Several events such as reperfusion of tissue, oxidative stress, and microglial activity can promote cell death in the penumbra region, which can lead to secondary neuronal death and further increased damage to stroke area [41]. These events can be triggered by a systemic inflammation, showing that the orodental diseases may aggravate the stroke condition [42].

Through targeted modification of a single risk factor or a cluster of multiple risk factors at the individual and community level, stroke prevention programs have been designed to decrease the incidence of stroke in the last decade [43,44]. There is much evidence about the association between stroke and oral health. However, the role of poor oral hygiene in the prevalence of stroke is controversial. For this, we chose to discuss the literature that was available for assessing the association of poor oral hygiene in stroke patients and the oral health of patients with stroke.

## 3. Orodental Health

The link between oral and overall health has long been established. Oral health refers to “a state of being free from oral infection, periodontal (gum) disease, tooth loss, tooth decay, oral and throat cancer, and other disorders that limit a person’s ability to smile, speak, taste, and chew [45,46]. Oral health can be maintained by developing a good oral care routine and by knowing lifestyle risk factors [47]. There are several oral health indicators such as frequency of dental scaling, frequency of tooth brushings, number of dental caries and missing teeth. Some efforts have been made to measure or scientifically quantify dental health. The oral health status of patients is typically assessed in a subjective manner [48].

The most widely used measures of oral hygiene status include “Simplified Oral Hygiene Index, Plaque Index, Gingival Index, Community Periodontal Index and Treatment Needs, and Decayed Missing Filled Teeth Index” [49,50]. The assessment of oral health indices is difficult and should be performed by trained dental professionals even though these indices provide uniformity. It is a well-known fact that the oral environment is one of the important sources of microorganisms. In addition to the local protective role, the oral cavity has a systemic role against pathological agents [51,52]. It has been estimated that over 500 bacterial species exist in the oral cavity, most of which are normal flora that plays a critical role in controlling the balance between bacterial species and protecting the oral cavity against the colonization and growth of external microorganisms. The composition of bacterial flora of the healthy oral cavity varies depending on location [53]. Saliva protects the mouth from microbes that lead to disease by washing away food particles and neutralizing the acids produced by bacteria. In the oral environment, rich in saliva and antimicrobial peptides, microorganisms are able to attach to host surfaces and start biofilm formation [54].

Oral infections include gingivitis, periodontitis, caries, implant-related, and oral mucosal infections and these are closely associated with daily living conditions and categorized as lifestyle-related diseases. Gingivitis is an inflammation of the gums triggered by bacterial microorganisms. If left untreated, it can progress to more serious stages of infection/inflammation called periodontitis [55]. Gingivitis and periodontitis may last for months or years and spread slowly the body when the bacteria enter the blood stream, leading to the release of a variety of inflammatory mediators [56,57]. Dental plaque, a unique biofilm, contains a wide variety of bacteria, viruses, and fungi [58]. Oral bacteria can be sub-classified according to their oxygen requirements. The surface of soft tissues is an ideal site for the colonization of aerobic bacteria and fungi, where oxygen levels are high at these areas. As oxygen levels are less than 1%, anaerobic bacteria appear to predominate in the gingival sulcus and periodontal pocket [59]. The species of oral bacteria vary considerably according to the stage of biofilm formation. Early colonizers found in the oral cavity are different species of streptococci and other natural microbiota, which have been not detected as respiratory bacterial pathogens [60]. In the later stages of colonization, biofilm microenvironment becomes anaerobic and ideal for more microbial species, such as *Tannerella forsythia*, *Treponema denticola*, *Fusobacterium nucleatum*, and *Porphyromonas gingivalis* to promote the risk of systemic infections [61]. Some of these bacteria have been implicated in cerebrovascular diseases such as *Streptococcus mutans* involved in dental caries, *P. endodontalis* as a key pathogen in endodontic infections, and *P. gingivalis* involved in gingivitis and periodontitis. *Streptococcus* species are detectable in all atherosclerotic plaque samples [62]. This mutant plays a critical role in accelerated onset of stroke in rats [63] as well as in patients who have had a hemorrhagic stroke [64]. The common and important viruses include cytomegalovirus, human papilloma virus, herpes simplex virus type 1 (HSV-1), and Epstein-Barr virus. Fungal infections in the oral environment are caused by Candida species, especially *Candida albicans*. In some cases, parasitic infections may also occur.

In 2016, several studies investigated the concept of the “brain-oral axis” to highlight the significant effect of oral health on the brain function [65,66]. Recently, the existence of an “oral-gut-brain axis” has also been suggested. The oral-gut communication is described from the constant swallowing of oral bacteria. The gut-brain communication is due to the bacterial-trigeminal nerve interaction. Therefore, the oral-brain and gut-brain axis are in a bi-directional relationship [67,68,69,70]. It was reported that frequent oral hygiene care has a negative connection, and infrequent oral hygiene behavior has a positive connection with the risk of stroke incidence [71]. Invasive oral pathogens can cause damage to the brain tissue directly or indirectly, resulting in stroke, cerebral infarction, cerebral hemorrhage, and subarachnoid hemorrhage. Oral bacteria easily enter the systemic circulation and reach the basal location of the brain; therefore, the oral cavity is located in proximity to the brain for studying the pathology of cerebral diseases. The human experimental model offers the unique opportunity to examine the bacterial communities present in the oral cavity and the subsequent response in real time.

## 4. Relation between Gum Disease and Stroke

### Gingivitis/Periodontitis

**Figure 3:**
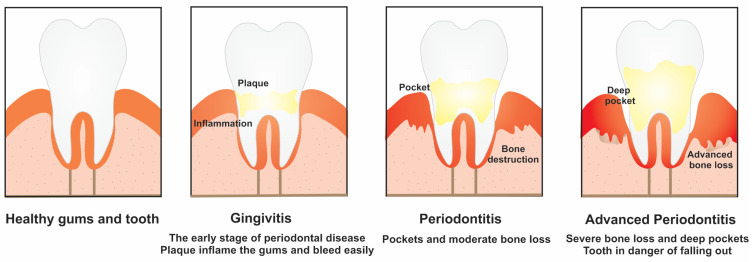
Gum disease: gingivitis and periodontitis.

Gingivitis is a mild form of gum disease that happens when bacterial plaque (dental biofilm) accumulates on the tooth. The symptoms of this disease are caused by chronic bacterial infection and inflammatory response of the host [72]. There are a combination of hereditary and environmental factors involved in the onset of gingivitis. If not treated, gingivitis can become an established inflammatory lesion (Figure 3).

The control of gingivitis is essential for the prevention of periodontitis. Among oral infections, periodontal diseases refer to inflammatory processes that affect the tooth-supporting structures such as the gingiva, alveolar bone, and periodontal ligament [73]. These events lead to tooth loss and contribute to systemic inflammation. Periodontitis is caused by local infections with periodontal pathogens, which in turn leads to systemic reactions, such as inflammation and immunological reactions [74]. The prevalence statistics of periodontal disease depends on the number of teeth, the area probed, sampling and possible bias, and case misclassification. Poor oral hygiene, smoking, diabetes, medication, stress, age, and hereditary factors are the most important risk factors for periodontal diseases. Several studies reported that gingivitis and periodontitis are more common among males as compared with females probably because of poor oral health habits in men and less frequent dental visiting [75,76].

It was reported that gum disease treatment alongside other stroke risk factors can decrease the risk of developing stroke. In early stages of gingivitis, the gums become red, swollen, and inflamed, reducing the blood and oxygen flows to the brain [77]. There is a higher prevalence rate of stroke and a lower survival rate of stroke in patients with periodontal diseases than patient with gingivitis during a follow-up period of 10 years [78]. The relationship between periodontitis and cerebrovascular accident was first investigated in 1963 by Mackenzi and Milard [79] but an in-depth understanding of the issue and its importance was not reported.

Periodontal pathogens can induce local and systemic inflammatory and immune reactions. Systemic inflammatory reaction has been reported to be higher in patients with periodontitis as compared to patients without periodontitis, thereby suggesting a strong association with ischemic stroke. Periodontal disease is associated with increased amount of circulating levels of inflammatory markers that are themselves indicators of stroke risk.

Microorganisms from the periodontal pockets can reach the systemic circulation during daily events such as eating, chewing, and tooth brushing [80]. Recent hypotheses suggest the mechanism of association between periodontal disease and stroke risk. Some bacterial species present in the oral cavity such as *Prevotella intermedia* and *P. gingivalis* and their released endotoxins may directly invade the epithelium and the endothelium [81]. Bacteria may also penetrate via the root canal of infected tooth or through periodontal pocket and ultimately enter the bloodstream to induce systemic inflammation [82,83]. According to the cytokine hypothesis, inflammatory cytokines secreted by the immune cells play a key role in vascular inflammation and injury [84]. The autoimmunization hypothesis explains the role of heat shock proteins (HSP65) expressed by the oral pathogenic bacteria in autoimmune reactions [85]. Besides, virulence factors associated with the pathogenicity of these bacteria may promote atherosclerosis leading to the deposition of cholesterol, cholesterol esters, and calcium into the walls of blood vessels. Unstable atherosclerotic plaques enclose a large lipid core, a thin fibrous cap, and macrophage-rich regions. The release of debris and thrombi from ruptured atherosclerotic plaques may result in distal embolization and stroke [86].

Oral bacterial DNA has been found in the coronary, carotid artery plaques, and arterial aneurysmal wall [87]. Patients with a severe form of periodontal disease presented higher bacterial loads in atherosclerotic plaques compared to those with medium periodontitis [88] It was demonstrated that the periodontal disease condition triggers the production of inflammatory mediators and clotting factors, such as tumor necrosis factor-alpha, interleukin -1beta, C-reactive protein, and fibrinogen owing to vascular infiltration after bacterial invasion or a chronic inflammation of the periodontal tissues. These changes may activate a pro-atherogenetic response at different regions, including the blood vessels in the brain, and may have a strong role in the onset of stroke [89]. Periodontal disease may also increase vascular oxidation stress and proteolytic activities, resulting in the rupture or formation of atherosclerotic lesions [78].

*P. gingivalis*-targeted treatment strategies were reported to protect against ischemic stroke. Though various studies have been performed to address this relationship, there are still conflicting results in this regard. Many of the studies included in this paper demonstrated an association between oral diseases and stroke, and the risk is higher among patients with periodontal disease. Morrison et al. [3] have demonstrated an indistinctive association between periodontal disease and stroke. This association increased to 2.12% for fatal ischemia. The results of a recent meta-analysis study demonstrated that the risk of ischemic stroke is 2.8-fold higher in patients with periodontal conditions than those without periodontal conditions [90]. Hashemipour et al. [91] conducted a case-control study on 100 patients (42 males and 58 females) suffering from stroke as the case group, and 100 hospitalized patients (44 males and 56 females) as the control group. According to the results, a significant association was found between stroke and the periodontal index; however, there was no significant association between stroke and gingival index. Syrjänen and co-workers [92] first exhibited the possibility of the connection between periodontitis and stroke. Several included studies demonstrated that an independent association exists between periodontitis markers and stroke [93,94,95]. Six prospective and seven retrospective studies were conducted by Sfyroeras et al. [84] to examine the link between periodontal disease and stroke. Subjects with periodontitis had a 1.47 and 2.63 times higher risk of stroke than subjects without periodontitis in prospective studies and in retrospective studies, respectively. It was reported that men with periodontal disease are almost 3 times more likely to develop stroke, compared with men without periodontal disease [79]. Although some authors reported a positive link between gum disease and stroke, no association or non-significant connection has been reported by others [96,97].

## 5. Relation between Tooth Conditions and Stroke

### 5.1. Dental Caries

Dental caries is one of the most common oral infections, and is characterized by the destruction and de-mineralization of the tooth [88]. Today, regular dental care is the only real preventive strategy for dental caries. While both periodontal disease and dental caries are biofilm-mediated diseases, dental caries are a complicated and multifactorial process such as many chronic diseases such as diabetes, cancer, and cardiovascular disease, which is pathophysiologically distinguished from periodontal disease [98]. Dental caries are the results of the interaction of a wide variety of environmental, behavioral, and genetic risk factors [99].

In dental caries, the ability of oral bacteria to access the bloodstream is plausible and may occur through different mechanisms already identified for periodontal disease [100]. Root canal space or marginal periodontium may serve as a direct communication pathway between oral microbiota and the direct systemic circulation. However, pathogens and/or host factors can enhance dental caries and the likelihood of the systemic spread of oral microbes [101]. In 2010, untreated caries in the permanent tooth was the most common health condition, affecting 35% of people worldwide. Despite the efforts to promote oral health through daily teeth brushing and using fluoride, dental caries are still known as one of the most prevalent oral diseases.

Since stroke and dental caries are common, examining their association could update the evidence on the link of stroke and dental health. Past studies have reported an association between dental infection and stroke incidence, but few studies have looked into what role dental caries might play. According to a new study, Sen et al. [100] examined the presence of dental caries in 6326 participants without prior stroke. According to the results, subjects with ≥1 coronal dental caries were at a higher risk for ischemic stroke incidence, compared with subjects without dental caries. The connection remained significant after controlling for race, gender, age, education, body mass index, diabetes, smoking status, and the presence of periodontal disease. Further, they found a potent and independent relationship between dental caries and the cardioembolic stroke subtype. In another cohort study performed by Chang et al. [71] the number of dental caries (≥4) was positively associated with the risk of occurrence for stroke. However, the link of dental caries with stroke remains controversial, and a study did not find any relationship between dental caries and transient ischemic attack or acute ischemic stroke [102]. Another research work was conducted to evaluate oral health status, salivary gland function, enzymatic and non-enzymatic antioxidant defense, and the oxidative damage to proteins and lipids in the saliva of patients post stroke. It was concluded that enzymatic and non-enzymatic systems were disturbed and oxidative damage to proteins and lipids was increased [103].

### 5.2. Tooth Loss

Tooth loss is an age-related and frequently-occurring disease. Although trauma or intentional tooth extraction can also lead to tooth loss, the primary cause of tooth loss is oral diseases [104]. Untreated dental and periodontal infections will eventually result in intentional or eventual tooth loss. Therefore, the risk of tooth decay and tooth loss can be decreased by good oral and dental hygiene including frequent tooth brushing and regular dental visits [105]. Since periodontal disease is the main reason for tooth loss after age 40 years, tooth loss in the adult population can be used as a key determinant of periodontal disease, or exposure to chronic infection [106]. The total number of missing teeth may reflect the enhanced inflammatory status of person. According to a study, nearly 50% of patients with tooth loss had dental caries and 30% of those with tooth loss had periodontal disease [107]. In another work, 62% of patients with tooth loss had periodontitis and 20% of patients with chronic periodontitis had tooth loss [108].

The process of tooth loss begins when plaque and calculus accumulate on the teeth surface and leads to gingival recession and/or loss of periodontal attachment, which “loosen” the teeth from the socket (Figure 3). To the best of our knowledge, there are very limited studies to examine the effect of the different timings of periodontitis and tooth loss and on the incidence of ischemic stroke. Baseline tooth loss was reported as a significant contributor to the increased risk of stroke, whereas recent tooth loss demonstrated a weak connection [75].

Tooth loss has been debated as a potential marker of cerebral stroke. Some cross-sectional studies have addressed the fact that patients with a fewer number of teeth may be associated with increased risks of stroke or other systemic diseases [109,110,111]. On the other hand, if tooth loss is caused during the early years of life or is due to other factors such as caries or trauma, the patients might not have been affected by periodontal disease for the rest of their life [112,113]. In such conditions, negative health effects of poor nutrition and unhealthy eating patterns may contribute indirectly to an increased incidence of stroke [114,115]. Several studies have demonstrated the association of tooth loss with the occurrence of stroke. For example, Heitmann and Gamborg [116] examined the incidence of fatal and non-fatal cardiovascular disease, coronary heart disease, or stroke among 1474 men and 1458 women, aged 30, 40, 50, and 60 years during a follow-up period of 5–13 years. Over a median follow-up of 7.5 years, 86 subjects (38 women and 48 men) developed stroke. The edentulous subjects exhibited a significantly higher risk of stroke (>3-fold) than those with 26–32 teeth remaining. A similar relationship was found between men and women, more and less educated, as well as smokers and non-smokers. Another study performed by Choe et al. [117] aimed to evaluate the association between tooth loss and different subtypes of stroke during a follow-up period of 14 years, among Korean men and women (n = 867,256, aged 30–95 years). The resulting data demonstrated a graded relationship between higher tooth loss and a higher risk of ischemic and hemorrhagic stroke both in men and in women. Grau et al. [118] demonstrated that the number of teeth was significantly lower in stroke patients compared to the population controls. Abnet et al. [118] followed 29,584 healthy, rural Chinese adults aged 40–69 years at baseline, noting the association of tooth loss with the leading causes of mortality among smokers such as heart disease, cancer, and stroke. The reference category were individuals who had missing teeth less than or equal to or greater than the median number of missing teeth among other subjects of the same age at baseline. Throughout the 10–15 years of follow-up, tooth loss was associated with a significant risk of stroke death, independent of smoking. Joshipura [119] demonstrated that men with <25 teeth had an increased risk of ischemic stroke than men who had ≥25 teeth. Wu and co-workers demonstrated a lower hazard ratio of 1.37 for stroke in subjects with periodontitis and 11 or more tooth loss compared with subjects with no gingivitis, periodontitis, or tooth loss [120].

However, several studies have reported no potential link between the number of teeth and stroke. Joshy et al. [121] demonstrated that tooth loss is not associated with stroke incidence among individuals aged 45–75 years. Morrison and co-workers [122] also detected a non-significant relationship between total fatal stroke and edentulousness, but they could not address ischemic stroke separately. A list of studies reviewed for knowledge regarding the relationship between stroke and orodental health is shown in Table 1.

### 5.3. Endodontic Lesions

Endodontic infections and their short- and long-term effect on overall health have been underway over the years. In its healthy state, the root canal system is free from infection and has no commensal microbiota. Therefore, any microorganism found in the root canal system can be considered as a potential pathogen [133,134]. Endodontic infections typically happen when dental caries extend deep into the root canal and the microbial pathogen reaches the apex of the teeth’s root, resulting in periapical lesions: apical periodontitis [135]. Bone loss around the periapical region is expected as the endpoint of an overactive immune response to the inflammatory condition of endodontic origin. Failure of the endodontic treatment can potentially be used for predicting the onset of apical periodontitis [136]. Despite differences in their origins, periodontitis and endodontic diseases demonstrate remarkable similarities such as a common microbiota that contains gram-negative anaerobic bacteria and elevated concentrations of systemic cytokines and inflammatory markers in association with both diseases [137]. Therefore, it has been proposed that endodontic lesions are more likely to develop cardiovascular disorders in a similar fashion to that found for periodontitis [138].

Bacteria derived from the infected root canal and also byproducts of systemic inflammation can affect major arteries and thus cause changes that promote the incidence of cardiovascular diseases [139]. The microorganisms associated with the endodontic infections are a selection from the oral commensal microbiota. *P. endodontalis* is an important gram-negative pathogen found in endodontic infections [140]. It is capable of invading vascular endothelial and smooth muscle cells and therefore interfering in endothelial function, which can in turn promote the risk of cardiovascular diseases [141]. Lipopolysaccharide is a major component of the outer membrane of gram-negative bacteria that elicit the production of pro-inflammatory markers. LPS was demonstrated to be an independent risk factor associated with the cardiovascular diseases [142]. The majority of studies have investigated the connection between endodontic lesions and cardiovascular diseases [143,144]. Pussinen et al. [145] suggested that the presence of *P. gingivalis* increases the risk of ischemic stroke. *P. gingivalis* is able to invade endothelial cells and promote the expression of adhesion molecules by endothelial cells. It can enhance monocyte and macrophage infiltration into the blood vessel wall, leading to chronic inflammation [146].

## 6. Effect of Orodental Treatment

Oral health care includes a specific set of medically necessary services for the prevention, primary diagnosis, and treatment of oral and dental diseases [147,148]. It has been demonstrated that invasive dental treatment for periodontitis may lead to the short-term disruption of the blood flow [149]. Invasive dental procedures may contribute to a transient rise in the risk of stroke. Epidemiological data also suggest that people have a higher risk of developing stroke for a few weeks after an oral surgical procedure. Invasive dental treatments and oral surgery favor bacterial dissemination from the oral cavity into the bloodstream, which trigger systemic inflammation and induce a state of acute vascular dysfunction [150].

In this regard, Minassian et al. [151] evaluated the risk of myocardial infarction and ischemic stroke in periods of time immediately after invasive dental treatment compared with the risk in all other time intervals. They demonstrated that invasive dental treatment had an increased risk for the incidence of vascular events in the first 4 weeks and gradually returned toward baseline levels by 6 months. A positive link was observed after exclusion of patients with hypertension, diabetes, or coronary artery disease or patients taking antiplatelet medications for a period before treatment. In another work, Li et al. [78] investigated the relationship between periodontitis with and without specific treatment and the incidence of ischemic stroke based on data from the Taiwan National Health Insurance Research Database 2005. Their study demonstrated that dental scaling and the intensive treatment for periodontal disease can help to reduce the risk of developing stroke, while patients who undergo tooth extraction therapy have a higher risk of stroke. Infections with theodontogenic origin are among the most common infections of the oral cavity, which can be treated by endodontic therapy, tooth extraction, or surgical treatment. In contrast, a study was conducted by Chen et al. [127] to estimate the risk of myocardial infarction and ischemic stroke in the Taiwanese population using the case-crossover (123,819 myocardial infarction patients and 327,179 ischemic stroke patients) and self-controlled (117,655 myocardial infarction patients and 298,757 ischemic stroke patients) case series design. The resulting data from both study designs demonstrated non-significant association between the invasive dental treatments, and the incidence of ischemic stroke or the risk ratio was close to unity. However, these results cannot exclude that orodental diseases may cause a long-term risk of ischemic stroke.

Poor oral hygiene is often detected to co-exist in most patients with oral cancer. Oral cancers are cancers found in the lip, other parts of the mouth, and the back of the throat. Apart from defined etiological factors such as alcohol and tobacco, other factors such as poor nutrition [152], chronic mucosal trauma [153], and poor oral hygiene [154] may contribute to oral cancer. Several studies have demonstrated poor oral hygiene as a risk factor for causing oral cancer [155,156,157].

However, there are limited data regarding the relative risks of ischemic stroke in patients with oral cancer. Wu et al. [158] demonstrated a connection between oral cancer and elevated risk of ischemic stroke among oral cancer patients treated with and without radiotherapy or chemotherapy, or both. This study included 21,853 patients detected with oral cancer from 2000 to 2008. Patients treated with radiotherapy, chemotherapy, or both had a 1.24-fold and 1.024 greater risk of ischemic stroke than those who underwent surgery alone and surgery plus chemotherapy and radiation therapy, respectively. The resulting data demonstrated a 0.23-fold lower incidence of ischemic stroke in the matched control group than in the oral cancer cohort. On the other hand, the risk of developing ischemic stroke was 2.77-fold greater for patients who received radiotherapy/radiotherapy/radiotherapy-radiotherapy and were aged < 40 years old than age-matched patients who received surgery alone. Perioperative stroke is a cerebrovascular incident that can arise intra-operatively. A study by Sukegawa et al. [159] described the case of a female patient who experienced peri-operative stroke. The authors demonstrated that there was no prophylaxis for the incidence of perioperative stroke in patients undergoing surgery for oral, neck, and head cancer. Therefore, the patient should be cautiously monitored for the early diagnosis of stroke. In another work, Chang and co-workers [160] evaluated the risk of stroke after surgery in oral cavity cancer patients. The resulting data demonstrated that neck dissection does not promote the risk of having a stroke. Patients are considered to be at high risk of a stroke based on their specific comorbidities, type of postoperative treatment, and older age. Functional improvement in patients undergoing rehabilitation can be influenced by oral health among hospitalized patients [161].

While this study explores the link between stroke and oral health status, it is not without its limitations. One of the limitations of this review is the complete reliance on previously published studies and the appropriateness of these studies with the criteria of the selection procedure. This paper is a clinically oriented narrative review and not a systematic review. We believe that PRISMA guidelines can improve the quality of the study and future research.

## 7. Conclusions

Prevention of stroke and other brain impairments is an urgent theme worldwide. Oral diseases have been linked with age-related disorders such as stroke, hence thebetter management of oral hygiene and dental health might lead to reduced stroke risk. Periodontal diseases and tooth loss are two common oral health measures. Gingivitis and periodontitis are preventable and treatable conditions. Lack of knowledge on the effects of poor oral health on systemic health together with the limited access to primary medical or dental care are considered to be partially responsible for the elevated risk of stroke, globally. There is a need for prospective studies to check the association between oral disease severity and increasing future stroke risk as well as to ascertain whether oral disease effects stroke survival. It seems important to further confirm potential associations between oral inflammatory conditions and stroke in large cohort studies employing state-of-the-art techniques to predict stroke as a function of oral health status, and biomarkers of systemic inflammation.

## Figures and Tables

**Table 1:** A summary of studies reviewed for knowledge regarding the link between stroke and orodental health.

Study Design	Oral Health Factor	Sample Size	Follow Up	Country	Stroke Subtype	Remarks	Reference
A cohort study	Periodontal disease	71 adults	4 years	Finland	Ischemic stroke	An association between poor oral health and acute ischemic stroke. Most patients had poor oral health and had 19.0 teeth left on median	[123]
A cohort study	Periodontal disease	5958 adults	17 years	US	Ischemic stroke	An independent association between severe periodontal disease and stroke	[124]
A retrospective cohort study	Periodontal disease	298,128 adults	10 years	Korea	-	Severe periodontal disease increased total stroke by 1.4%	[125]
A cohort study	Periodontal disease	15,792 adults with the age of 45–64	15 years	US	Cardioembolic and thrombotic stroke	Periodontal disease was significantly associated with cardioembolic and thrombotic stroke subtypes	[30]
A meta-analysis of cohort studies	Periodontitis, gingivitis, and tooth loss	-	-	-	Fatal or non-fatal, ischemic or hemorrhagic	Both periodontitis (relative risk 1.63) and tooth loss (relative risk 1.39) were associated with the stroke risk	[126]
A case-control study	Periodontitis and gingivitis	771 adults	-	Germany	Cerebral ischemia	Periodontitis was found to be an independent risk factor only in younger patients and men	[102]
A prospective cohort study	Severe periodontal disease	807 adults aged ≥40 years	4 years	Latin America	-	A direct but modest association between periodontal disease and stroke	[127]
A cohort study	Periodontal disease and tooth loss	3389 adults over 40 years of age	-	Korea	-	Loss of tooth due to periodontal disease is a risk factor for stroke. The stroke risk was 2.17 times higher in the group with less than 19 remaining teeth	[128]
A cohort study	Periodontal disease and tooth loss	41, 380 adults	4 years	US	Ischemic stroke	A modest association was found between baseline periodontal disease history and stroke. Men with ≤24 teeth at baseline were at a higher risk of stroke compared to men with ≥25 teeth	[75]
A cohort study	Number of dental caries and tooth loss	206,602 adults	10.4 years	Korea	Cerebral infarction, cerebral hemorrhage, and subarachnoid hemorrhage	Regular oral hygiene behavior was negatively and number of dental caries was positively associated with stroke occurrence	[71]
A dose-response meta-analysis study	Tooth loss	-	Updated to April 2017	-	-	A significant dose-response association between tooth loss and stroke risk	[129]
A review study	Tooth loss	-	10 years	-	-	A relationship between early tooth loss and the stroke occurrence	[130]
A cross-sectional study	Tooth loss	410,939 adults	4 years	US	Cerebral vascular accidents, cerebral infarctions, and cerebrovascular ischemia	Tooth loss had a potential association as an independent factor in the stroke incidence	[131]
A national, population-based, longitudinal study	Tooth loss	24,393 black and white adults aged 45 years and over	10 years	US	Stroke mortality	Tooth loss was positively associated with C-reactive protein, white blood cell count, and stroke/TIA	[132]

Notes: * United States.

## Data Availability

The raw/processed data can be shared by request from the corresponding author.
